# Supplementary material for: Procoagulant Activity of Umbilical Cord-Derived Mesenchymal Stromal Cells’ Extracellular Vesicles (MSC-EVs)
Source: Int J Mol Sci. 2023 May 24;24(11):9216. doi: 10.3390/ijms24119216 (PMC10252357; doi:10.3390/ijms24119216)
Supplement: Supplementary file 1 [file ijms-24-09216-s001.zip › supplemental materials and S1-S4 final submission 23 May 2023.pdf]

Procoagulant activity of umbilical cord-derived mesenchymal stromal cells' extracellular vesicles (MSC-EVs)

Supplemental material

Adrienne Wright [a,b], Orman (Larry) Snyder [a,b], Hong He [a,b], Lane K. Christenson [c], Sherry Fleming [d], Mark L Weiss [a,b,*]

[a] Department of Anatomy and Physiology, Kansas State University, Manhattan, KS 66506, USA;

[b] Midwest Institute of Comparative Stem Cell Biotechnology, Kansas State University, Manhattan, KS 66506, USA

[C] Department of Cell Biology and Physiology, University of Kansas Medical Center, Kansas City, KS 66160s USA;

[D] Division of Biology, Kansas State University, Manhattan, KS 66506, USA

CUC-MSC expansion

Canine umbilical cord-derived mesenchymal stromal cells (CUC-MSCs) were isolated, culture-expanded, characterized, and cryopreserved using a previously described protocol [1]. As shown schematically in Figure 4, cryopreserved CUC-MSCs (P1) were thawed and seeded at 2x10^4 cells/cm2 density on gelatin-coated tissue culture vessels. CUC-MSCs were allowed to recover from cryopreservation for one passage before use. CUC-MSCs were maintained in culture using previously described methods using ACB cell culture medium and incubated at 37°C, 90% humidity, and 5% CO_2 in a HeraCell 150i incubator [1]. Once cells reached ~80-90% confluency, cells were lifted using 1.75% Nattokinase for 30 minutes at 37°C [1]. A live/dead cell count was performed at passage using acridine orange/propidium iodide (AOPI) staining solution (Nexcelom Bioscience, Cat. No., CS2-0106-5ML) on a Nexcelom Auto 2000 Cellometer (immune cells, low RBC program, Nexcelom Biosciences, Lawrence, MA). MSCs were seeded at a density of 2x10^4 cells/cm^2 on two gelatin-coated flasks (i.e., one flask to maintain the cell line and one to produce CM). MSCs were maintained in culture through P12, and CM was collected at each passage from P2-P12.

Preparation of size exclusion chromatography column

A size exclusion chromatography (SEC) column was prepared as previously described using Sepharose CL-2B (GE Healthcare, Cat. No. 65099-79-8) [2,3]. Briefly, the tip of a 20 mL sterile syringe (EXELINT, Cat. No. 26280) was stuffed with cotton wool, and 20 mL of Sepharose CL-2B slurry was poured into the syringe [3]. The column was allowed to settle overnight at 4°C. After settling, the dimensions were 7.5 cm (length) x 2.21 cm (diameter). Next, the column was packed by passing three volumes of sterile, degassed Dulbecco's phosphate-buffered saline (DPBS, Gibco, Cat. No. 14-190-250) through at a gravity-controlled rate. The column packing quality and void volume were estimated by passing 0.25 mL of Blue Dextran (Sigma Aldrich, Cat. No. D5751) through the column. The void and separation efficiency was evaluated by layering 0.25 mL Blue Dextran or bovine serum albumin, respectively (BSA, Sigma Aldrich, Cat. No. A3912-500G). Fractions (0.25 ml) were collected after the void volume, and those containing EV or BSA were identified by protein concentration.

EV isolation by a combination of ultrafiltration and size-exclusion chromatography (SEC)

As previously described, EVs were isolated from CM using a combination of ultrafiltration and size-exclusion chromatography [3]. Briefly, CM was thawed from -80°C at room temperature and centrifuged at 3200 *g* for 30 minutes at four °C (Eppendorf 5810R centrifuge, swing bucket rotor A-4-62, Cat. No., FL08517291) to pellet cells and cell debris. The supernatant was filtered through a 0.22 µm syringe filter (Fisherbrand, Cat. No. 09-720-004) and then concentrated by approximately 100x using an Amicon Ultra-15 filter unit with an Ultracel-100 membrane (MWCO = 100 kDa, Merck Millipore, Cat. No. UFC910024). The sample was centrifuged at 3200 *g* at four °C until the sample volume was ≤ 300 µL, and that sample was collected in a separate microcentrifuge tube. The membrane was jetted with 200 µL DPBS, and any adherent EVs were collected from the membrane and added to the sample tube. The concentrated sample was layered onto the SEC column and was eluted with sterile, degassed DPBS. Following the void volume, 27 fractions of 250 µL were collected. Aliquots were sampled to estimate protein concentration using a NanoDrop 8000 spectrophotometer (1.5 µL per reading, samples measured in technical triplicates, Thermo Scientific, Waltham, MA). The four fractions following the void volume were pooled and concentrated 4x using the Amicon Ultracel-100 membrane.

EV isolation by ultracentrifugation

EVs were isolated from CM by ultracentrifugation using a previously described method with slight modifications [4]. Briefly, CM was thawed from -80°C at room temperature and centrifuged at 3200 *g* for 30 minutes at four °C to pellet cells and cell debris. The supernatant was collected, filtered through a 0.22 µm syringe filter, and transferred to a 38.5 mL open-top polypropylene tube (Beckman Coulter, Cat. No., 326823). CM was centrifuged at 20,000 *g* for 30 minutes at four °C (Sorvall™ wX+ Ultra Series Centrifuge, Thermo Fisher Scientific) with a SureSpin 630/36 rotor (Thermo Scientific, Cat. No., 79368). Following the first spin, CM was transferred to a new polypropylene tube and centrifuged at 120,000 *g* for 90 minutes at 4°C. The supernatant was discarded, and the pellet was resuspended in sterile DPBS.

Dynamic light scattering (DLS)

DLS, zeta surface potential, and polydispersity index (PDI) were used to analyze the hydrodynamic size distribution, surface charge properties, membrane integrity, and

overall EV stability, as previously described [3,4]. Measurements were made using the Zetasizer Nano ZS (Malvern Pananalytical., Malvern, United Kingdom). Instrument settings were ten runs of 10 seconds with three repetitions per sample. Six independent samples from each passage were analyzed. Measurements were made in technical triplicates and averaged for comparison. PDI and zeta surface potential were compared using two-way repeated measures ANOVA with isolation and passage as factors. After significant ANOVA main or interaction terms were found, pre-planned pairwise comparisons were performed using the Holm-Sidak method. DLS size was compared using the Mann-Whitney rank sum test.

Transmission electron microscopy (TEM)

TEM was performed to visualize EV morphology and estimate EV size as previously described [3,4]. Lyophilized samples were rehydrated using sterile DPBS. Masked samples were prepared, and images were collected at the University of Kansas Medical Center. Approximately 62.1 μm^2 was sampled, and at least 18 micrographs were collected per sample. EV size was estimated by measuring data from two independent lines chosen randomly at each isolation x passage group (i.e., early and late). Extreme ends of the passage groups were selected (P2 and P12) since we assumed those to show the most significant differences. From this data, sizes were averaged by the isolation method and passage group to generate size measurements to be compared using the Mann-Whitney rank sum test.

Protein

The protein concentration of samples isolated by ultracentrifugation was determined using a Pierce BCA protein assay kit (Thermo Scientific, Cat. No. 23225) according to the manufacturer's instructions. As previously described, the protein concentration of samples isolated by SEC was determined using a Pierce micro BCA protein assay kit (Thermo Scientific, Cat. No. 23235) [3,4]. Samples were plated in triplicate in a 96-well plate (Corning, Cat. No. 3370). Protein standard curves were generated using albumin standards provided with each respective kit and by averaging the technical triplicate wells. The absorbance was read at 562 nm using a SpectraMax i3x plate reader (Molecular Devices, San Jose, CA) according to the manufacturer's instructions. DPBS was used as a blank for background subtraction. Individual sample protein measurements were made from the averaged triplicate readings. Post-lyophilization content for each

sample was calculated as the average of triplicate readings measured the absorbance at 280 nm on a NanoDrop 8000 spectrophotometer with distilled water as the blank for background subtraction. Protein concentration was averaged among isolation methods and compared using the Mann-Whitney rank sum test.

Dot blot

A polyvinylidene fluoride (PVDF) membrane (Merck Millipore Ltd., Cat. No. IPVH0010) was activated by submerging it in methanol for 30 minutes. Lyophilized EV samples were rehydrated using 100 µL sterile water. Post-lyophilization protein content was measured as described above, and 0.75 µg of protein was blotted onto the membrane and air-dried. Water and canine MSC whole cell lysate was used as negative and positive controls, respectively. With gentle rocking, the membrane was blocked with 5% non-fat dried milk solution for 30 minutes at room temperature. The membrane was probed with primary antibodies anti-CD9, anti-CD63, anti-CD81, anti-ALIX, anti-CD142, and anti-β-actin (Supplemental Table 2) overnight at four °C with gentle rocking. The following morning, membranes were washed three times using TBST buffer (tris-buffered saline with 0.1% Tween-20 detergent) and incubated with secondary antibodies (see Table 2) for one hour at room temperature with gentle rocking. Post-incubation, strips were washed three times with TBST buffer. Chemiluminescence detection reactions were performed using SuperSignal West Femto substrate (Thermo Scientific, Cat. No., 34095) according to the manufacturer's directions. Images were captured using a Kodak Image Station 4000 after 2 minutes of exposure. Each sample was blotted for five proteins: CD9, CD63, CD81, ALIX, and TF.

Nanoparticle tracking analysis (NTA)

As previously described, NTA was used to estimate the EV population size distribution and concentration using a NanoSight LM-10 (Malvern Pananalytical Ltd., Malvern, UK) [3,5]. Briefly, the machine was calibrated using 50 nm and 100 nm size calibrated standards (Malvern Pananalytical Ltd., Cat. Nos., NTA4087, and NTA4088, respectively). Measurements were made at a constant temperature of 25°C ± 1°C to ensure constant viscosity of samples. Sterile DPBS was used as a negative control. Samples were unfiltered. Sample dilutions ranged from 1:5 to 1:100 in NanoPure water (Barnstead/Thermolyne Nanopure lab water system) so that samples were in the desired

range of 30-50 particles/frame. Acquisition settings were constant: blue 405 nm laser, camera type scientific CMOS, camera level 13, and detection threshold 3. Nanosight software (NTA 3.3) analyzed 60-second videos with five repetitions per sample. The concentration measurement reported was made from the five technical replicates and averaged for comparison via two-way repeated measures analysis of variance (ANOVA), with factors being passage (P2-P5; P9-P12) and isolation (UC and SEC).

Procoagulant assay

The procoagulant activity of EVs isolated from canine UC-MSCs CM was assessed using a protocol proposed by Che et al. with modifications [6]. 5x10^7 EVs per well were diluted in HEPES buffer (10 mM HEPES, 137 mM sodium chloride, five mM calcium chloride, four mM potassium chloride, ten mM glucose, 0.5% bovine serum albumin, pH 7.4) to a final volume of 50 µL. Diluted EVs were incubated with 1 nM FVIIa (Haematologic Technologies, Cat. No., HCVIIA-0031) and ten µg/mL FX (Haematologic Technologies, Cat. No., HCX-0050) for 15 minutes at 37°C. Following incubation, a chromogenic substrate for FXa activity (40 µM, Chromogenix S-2765, Diapharma, Cat. No., S821413) was added, and the color change was measured at 405 nm every minute for 20 minutes using a SpectraMax i3x plate reader. The amount of FXa generated was calculated based on a standard curve of purified FXa (Haematologic Technologies, Cat. No., HCXA-0060). All samples were loaded into a 96-well plate in technical triplicates, and the absorbance readings were averaged. HEPES buffer served as a blank for background subtraction. To account for TF-independent FXa generation, control wells (EVs and FX only) were subtracted. MSC whole cell lysate and FX with FVIIa were positive and negative controls, respectively. To test TF-mediated FXa generation, EVs were incubated with anti-CD142 (Invitrogen clone HTF-1, functional grade, Cat. No., 16-1429-82 or Bioss polyclonal, Cat. No., BS-4690R) for 1 hour at four °C before incubation with FVIIa and FX. Six independent cell lines were analyzed at early (P2) and late (P12) passages.

Supplemental Discussion

Multiple size measurement methods create differences in measured size (as might be expected)

NTA, DLS, and TEM estimated EV size. NTA and DLS agreed that EV size was affected by the isolation method but not the passage. Both approaches provided size estimates within the desired exosome range (30-150 nm) [7-11]. The DLS size estimate of SEC-isolated EVs extended beyond the exosome range at 170.9 nm. However, this value is similar to other reports for MSC-EVs [4,12]. EVs isolated via SEC were significantly larger than those isolated via UC. We previously reported [3,4] DLS size estimates for human MSC-derived EVs (-80ºC storage group) separated via SEC were 165 nm. Our results support the notion that the UC isolation co-isolates smaller particles and is supported by the aggregates observed in TEM (as previously reported [13]).

No size differences were detected between Early and Late passage EVs by either DLS or NTA, indicating that the population of EVs isolated does not accumulate a subpopulation of larger vesicles (e.g., apoptotic bodies) with increasing time in culture, similar to previous reports [14,15]. Taken together with the particle concentration data, this data shows that although fewer EVs were isolated from late passage CM, the EV population isolated is similar in size. In contrast, TEM data alluded to larger-size EVs isolated from late passage CM with no difference between isolation methods. NTA and DLS data were obtained and averaged from every sample, but TEM data was performed on only two randomly-chosen MSC lines at each passage group and isolation method. Furthermore, the TEM data were collected using samples that had been lyophilized and reconstituted. It is possible that the lyophilization process shifted EV size due to osmotic or surface charge-related effects or due to changes in protein content in Later passage EVs. The sample size for TEM data is small, with less than 50 vesicles sampled per experimental condition. Therefore, care must be exercised when comparing the size estimates obtained

by TEM with those from DLS or NTA. This is a limitation of this work. That said, the TEM size estimates agree with the size estimates provided by NTA. In future work, a more extensive sampling (e.g., > 100 vesicles/ experimental condition) would generate better TEM size estimates and may resolve questions raised by other observations (i.e., CD63 staining and changes in protein concentration).

Effect of extensive expansion and isolation method on EV yields

MSC-EV yield was affected by time in culture such that later passage cells produced lower EV yields, and a negative trend was observed between EV yield with cumulative population doublings. When EV release from MSCs per cell was estimated, a similar negative trend was observed. Five of the six MSC lines grew consistently through P12, and one line (CUC30) showed signs of slowing. The results support the notion of culture expansion affecting MSC and MSC-EV physiology. The only apparent difference was the loss of CD63 expression in EVs at late passages. The field generally considers minimal expansion of MSCs, such as those in the early passages group here, for clinical trial applications.

EV yield was affected by the isolation method employed. Here, UC was more efficient (i.e., yielded higher nanoparticle counts per mL) than SEC. Still, protein concentration in UC samples was 10x higher than SEC, and protein aggregates were observed in UC-isolated EV samples in TEM. In contrast to our results, previous work reported that SEC yields more EVs than UC [16,17]. Still, others said no differences between the EV yield of UC and SEC [18]. Thus, the literature needs to be more consistent. These could be attributed to variations in isolation protocols or EV sources. We speculate that protein aggregates in the UC sample affect NTA particle per mL, contributing to a significantly smaller population. EV size estimates support this supposition: SEC yielded a larger size in NTA and DLS than UC. Our results suggest that UC-only-based EV isolation contains protein aggregates, not more EVs.

Protein content was affected by the isolation method: SEC had an order of magnitude lower protein concentration in the EV samples than UC. SEC had significantly more EVs per ug protein than UC when EV per ug protein was calculated. Culture factors may impact EV protein concentration, too. Late passages or higher CPD cells yield fewer EVs per ug protein than early passages, and a trend was observed. Similarly, the dot blot showed a loss of EV's CD63 staining associated with the late passages. The observations here agree with previous work indicating that SEC isolation reduces protein concentration in EV samples compared to UC alone [19,20]. We suggest that SEC isolation produces a more highly enriched or "pure" EV product, based upon the higher number of EVs per µg protein than the UC isolation method employed. Previous work indicated that the addition of density gradient UC could prevent the co-isolation of EVs with other proteins [21].

Efficiency by Design of Experiments (DoE) approach

Our DoE approach permitted estimates of the effects of culture (passage or CPD) and isolation method. Several general observations were made. First, CM collected in early passages produced higher EV yield (either nanoparticle per cell or nanoparticles per mL) than in late passages. Second, as seen by a negative linear relationship of EVs with CPD. When considering the variability of EV yield, we first evaluated the performance of MSCs to expand. There was good consistency in five of the six MSCs to grow over the 12 passages. We cannot explain why and have no reason to consider them statistical outliers. Thus, the small sample size is a limitation of our study. Third, the isolation method affected yield, and while UC was "more efficient" in that it produced more nanoparticles per cell, it appears to have greater variability than SEC. Both isolation methods had lower yields with increasing CPD. Fourth, there was a trend for nanoparticle yield per MSC to increase with an increased population doubling time. Again, the trend line is a preliminary observation and needs further confirmation since our dataset had a few slowly growing MSCs (e.g., > 100 hr PDT).

Regarding zeta potential, both cell passage and the EV isolation method affected zeta potential. SEC isolation method produced EVs with lower (more negative) zeta potential than UC, indicating an increased surface charge and perhaps greater membrane stability in SEC-isolated EVs than UC-isolated ones. Since UC may damage EV membranes and result in the formation of the small aggregates observed in TEM, the change in surface charge reflects membrane damage. Still, no direct evidence of EV membrane damage has been provided here. We cannot explain why the surface charge would change in the UC isolation method at later passages. We also noted that extended culture affects CD63 staining in dot blot but did not affect other dot blot or TF staining.

Our previous report [60] showed that pre-processing CM storage at -80ºC was used before EV isolation. CM was collected from six different canine umbilical cord-derived MSC lines selected randomly from a bank of over 30 canine MSC lines. MSCs were used from passages P2 to P12 and isolated using either SEC or UC per the DoE protocol. Because of the DoE protocol, multiple CM samples were collected daily. Due to COVID interruptions, there was insufficient time for CM samples to have EVs isolated within 24 hours, so pre-processing CM storage was critical to this work. In addition, pre-processing storage efficiently used shared equipment, and the maximum number of samples for the rotor (six samples) were processed at once. DoE increased experimental efficiency, minimized UC runs, and reduced the impact of batch-to-batch variability.

EVs Produced per MSC

EVs released per MSC were estimated using the same assumptions used previously [3]. Canine MSCs released an average of $2.02 \times 10^4 \pm 1.55 \times 10^4$ EVs per cell in the 24 h conditioning period. This is like the average of $2.19 \times 10^4 \pm 8.5 \times 10^3$ EVs per cell reported for human MSCs [3,22]. In contrast, Crain et al. reported an average of $5.78 \times 10^3 \pm 3.3 \times 10^3$ EVs per cell from canine WJ-MSCs. The disparity in EV yield is likely due to differences in medium conditioning period, canine MSC isolation/culture conditions, and EV isolation

protocol since Crain's team used a density gradient UC step that might decrease the efficiency of yield while increasing EV isolation "purity" [12]. In summary, our protocols produce canine EVs from early and late passage MSCs, which were comparable in number to human MSCs [3], suggesting that EV yield is similar between human and canine MSCs.

Linear regression revealed that nanoparticles released per cell were negatively correlated with extended time in culture. This relationship was seen by plotting EV yield versus cumulative population doublings since the consensus is that cumulative population doublings are more comparable across laboratories than passage [23]. As a cell accumulates population doublings, the number of EVs released per cell decreases. This data indicates that early passage cells are more efficient for EV production. Additionally, the protein content of EVs appears to increase in a later passage, and TF staining and CD63 staining in dot blot seem to change, too. These findings may have implications, specifically when considering translation to clinical applications requiring large numbers of EVs. While a functional assay for EVs was recently suggested [24], the practical impact of MSC culture on EV and MSC function was not assessed here. This is a limitation of the present study.

It is accepted that late passage or "aging" MSCs display increased senescence markers, decreased differentiation potential, decreased clonogenicity, potentially accumulated genetic mutations, morphological-feature changes, and different phenotypes in culture [25-29]. Aged MSCs might refer to culture-related aging, such as higher cumulative population doublings or MSCs collected from older-age donors. Regarding the present work, considering that UC-MSCs derive from consistently aged and young donors, aging refers to aging resulting from time in culture. In this regard, Zhuang et al. reported that late-passage MSCs (P15) display more robust immunosuppressive properties than early-passage MSCs (P3) [28]. The Phinney laboratory has correlated the loss of TWIST1 with the immunosuppressive properties of MSCs [30]. Here, we noted that EV protein

content increased with time in culture regardless of the EV isolation method employed, and TF dot blot staining intensity tended to increase. In future work, the TF and immunosuppressive properties of MSCs and MSC-derived EVs should be evaluated in the context of developing an optimized expansion phase for clinical scale-up for MSCs or MSC-derived EVs.

PDI describes the heterogeneity of the size distribution of a given particle population [31]. Samples with a PDI value greater than 0.7 indicate a wide size range and, thus, are not ideal for analysis by DLS [31]. All samples here were below the 0.7 threshold and were similar to those reported previously [3]. PDI did not differ between early and late passage EVs (0.456 vs. 0.479, respectively). This data, taken together with the NTA and DLS size estimates, confirms that EVs derived from late passage EVs are not co-isolated with other larger-sized vesicles. PDI did differ between UC and SEC isolation. UC samples displayed more homogeneity, indicated by a PDI value of 0.381 compared to 0.554 for SEC-isolated EVs. This was unexpected because sample purity is a concern when isolating EVs using UC methods, as indicated by our TEM results. We hypothesize that since PDI is directly related to size, the smaller PDI value in UC was related to the smaller hydrodynamic size in UC samples.

Zeta potential was used to measure EV surface charge, colloidal stability, and membrane integrity [32]. Like human MSC-EVs and previous reports, canine MSC-EVs displayed a negative zeta potential [4,22]. ANOVA's main effects (passage and isolation) and a significant interaction term were observed here. We will discuss the interaction term and not the main effects. For passage within the isolation method, there were opposite trends. For SEC-isolated EVs, as passage increased, the zeta potential of EVs remained constant. For UC-isolated EVs, as passage increased, the zeta potential of EVs changed. This suggests that the combination of later passage and UC impacts the stability and integrity of EVs. Alternatively, UC may disrupt EV membranes, and later passage EVs are

more susceptible to damage. For isolation within a passage, the zeta potential of EVs differed, and SEC-isolated EVs had a more negative zeta potential alluding to higher membrane stability and integrity of EVs. Again, this data suggests that UC may reduce EV membrane stability. This finding is important because the effects on the EVs from being sedimented against a solid surface are unknown, but it is thought to damage or compromise their contents [33,34].

Here, EVs isolated via UC had a 10-12x higher protein concentration than samples isolated using SEC, similar to previous reports [16,35]. This suggests significant co-isolation of proteins and lipoproteins with the UC protocol and points out how adding density gradient UC could potentially improve the purity of EVs [36,37]. As reported by Brennen et al., the purification of EVs from soluble proteins was calculated as the particle number divided by the amount of protein in the sample [19]. SEC samples produced a median of 2.9×10^9 particles per µg of protein compared to 3.3×10^8 particles per µg for UC samples (approximately 10x more EVs with SEC per ug protein). This is similar to previous reports for EVs isolated from human serum and CM of iPSC cells [16,19]. This makes a case that SEC EV yields are of higher purity than UC samples. Density gradient UC improves EV isolation, and adding this step might increase EV purity [36-38]. That question was not evaluated here.

Dot blots for exosome proteins, including tetraspanins (CD9, CD63, and CD81) and ALIX, were used to characterize EVs. Since EV samples isolated via SEC had low protein concentration, EV samples were lyophilized and reconstituted to increase protein concentration 10x. This allowed us to load less volume onto the PVDF membrane, producing more appealing dot blots. All EV samples were stained for CD9, CD63, CD81, and Alix, similar to previous reports [3,4,39]. This data demonstrates the enrichment of exosome proteins using both isolation methods. Expression of exosome marker CD63 was not detected in EVs derived from late passage MSCs, regardless of the isolation method.

Canine MSC-EVs started to lose expression of CD63 by P5 (data not shown), and it was undetectable by P12.

Moravcikova et al. said that MSC CD63 surface expression initially increased with passage and then decreased at passage 5 [100]. Similarly, Patel et al. reported reduced levels of CD63 after five serial passages of MSCs [36,83]. We need information that might reconcile these differences.

References

1. **Wright, A.; Snyder, L.; Knights, K.; He, H.; Springer, N.L.; Lillich, J.; Weiss, M.L. A Protocol for the Isolation, Culture, and Cryopreservation of Umbilical Cord-Derived Canine Mesenchymal Stromal Cells: Role of Cell Attachment in Long-Term Maintenance. *Stem Cells Dev* 2020, *29*, 695-713, doi:10.1089/scd.2019.0145.**
2. **Boing, A.N.; van der Pol, E.; Grootemaat, A.E.; Coumans, F.A.; Sturk, A.; Nieuwland, R. Single-step isolation of extracellular vesicles by size-exclusion chromatography. *J Extracell Vesicles* 2014, *3*, doi:10.3402/jev.v3.23430.**
3. **Wright, A.O.L.S.C., L.K; He, H.; Weiss, M.L. Effect of pre-processing storage condition of cell culture conditioned medium on extracellular vesicles derived from human umbilical cord-derived mesenchymal stromal cells. *International Journal of Molecular Sciences* 2022, *23*, doi:https://doi.org/10.3390/xxxxx.**
4. **Abello, J.; Nguyen, T.D.T.; Marasini, R.; Aryal, S.; Weiss, M.L. Biodistribution of gadolinium- and near infrared-labeled human umbilical cord mesenchymal stromal cell-derived exosomes in tumor bearing mice. *Theranostics* 2019, *9*, 2325-2345, doi:10.7150/thno.30030.**
5. **Snyder, O.L.; Campbell, A.W.; Christenson, L.K.; Weiss, M.L. Improving Reproducibility to Meet Minimal Information for Studies of Extracellular Vesicles 2018 Guidelines in Nanoparticle Tracking Analysis. *J Vis Exp* 2021, doi:10.3791/63059.**
6. **Che, S.P.Y.; Park, J.Y.; Stokol, T. Tissue Factor-Expressing Tumor-Derived Extracellular Vesicles Activate Quiescent Endothelial Cells via Protease-Activated Receptor-1. *Front Oncol* 2017, *7*, 261, doi:10.3389/fonc.2017.00261.**
7. **Barros, F.M.; Carneiro, F.; Machado, J.C.; Melo, S.A. Exosomes and Immune Response in Cancer: Friends or Foes? *Front Immunol* 2018, *9*, 730, doi:10.3389/fimmu.2018.00730.**
8. **Colombo, M.; Raposo, G.; Thery, C. Biogenesis, secretion, and intercellular interactions of exosomes and other extracellular vesicles. *Annu Rev Cell Dev Biol* 2014, *30*, 255-289, doi:10.1146/annurev-cellbio-101512-122326.**
9. **Hessvik, N.P.; Llorente, A. Current knowledge on exosome biogenesis and release. *Cell Mol Life Sci* 2018, *75*, 193-208, doi:10.1007/s00018-017-2595-9.**

10. Kalra, H.; Drummen, G.P.; Mathivanan, S. Focus on Extracellular Vesicles: Introducing the Next Small Big Thing. *Int J Mol Sci* 2016, *17*, 170, doi:10.3390/ijms17020170.
11. Silva, A.M.; Almeida, M.I.; Teixeira, J.H.; Maia, A.F.; Calin, G.A.; Barbosa, M.A.; Santos, S.G. Dendritic Cell-derived Extracellular Vesicles mediate Mesenchymal Stem/Stromal Cell recruitment. *Sci Rep* 2017, *7*, 1667, doi:10.1038/s41598-017-01809-x.
12. Crain, S.K.; Robinson, S.R.; Thane, K.E.; Davis, A.M.; Meola, D.M.; Barton, B.A.; Yang, V.K.; Hoffman, A.M. Extracellular Vesicles from Wharton's Jelly Mesenchymal Stem Cells Suppress CD4 Expressing T Cells Through Transforming Growth Factor Beta and Adenosine Signaling in a Canine Model. *Stem Cells Dev* 2019, *28*, 212-226, doi:10.1089/scd.2018.0097.
13. Linares, R.; Tan, S.; Gounou, C.; Arraud, N.; Brisson, A.R. High-speed centrifugation induces aggregation of extracellular vesicles. *J Extracell Vesicles* 2015, *4*, 29509, doi:10.3402/jev.v4.29509.
14. Kadota, T.; Fujita, Y.; Yoshioka, Y.; Araya, J.; Kuwano, K.; Ochiya, T. Emerging role of extracellular vesicles as a senescence-associated secretory phenotype: Insights into the pathophysiology of lung diseases. *Mol Aspects Med* 2018, *60*, 92-103, doi:10.1016/j.mam.2017.11.005.
15. Takasugi, M.; Okada, R.; Takahashi, A.; Virya Chen, D.; Watanabe, S.; Hara, E. Small extracellular vesicles secreted from senescent cells promote cancer cell proliferation through EphA2. *Nat Commun* 2017, *8*, 15729, doi:10.1038/ncomms15728.
16. Nordin, J.Z.; Lee, Y.; Vader, P.; Mager, I.; Johansson, H.J.; Heusermann, W.; Wiklander, O.P.; Hallbrink, M.; Seow, Y.; Bultema, J.J.; et al. Ultrafiltration with size-exclusion liquid chromatography for high yield isolation of extracellular vesicles preserving intact biophysical and functional properties. *Nanomedicine* 2015, *11*, 879-883, doi:10.1016/j.nano.2015.01.003.
17. Takov, K.; He, Z.; Johnston, H.E.; Timms, J.F.; Guillot, P.V.; Yellon, D.M.; Davidson, S.M. Small extracellular vesicles secreted from human amniotic fluid mesenchymal stromal cells possess cardioprotective and promigratory potential. *Basic Res Cardiol* 2020, *115*, 26, doi:10.1007/s00395-020-0785-3.
18. Gheinani, A.H.; Vogeli, M.; Baumgartner, U.; Vassella, E.; Draeger, A.; Burkhard, F.C.; Monastyrskaya, K. Improved isolation strategies to increase the yield and purity of human urinary exosomes for biomarker discovery. *Sci Rep* 2018, *8*, 3945, doi:10.1038/s41598-018-22142-x.
19. Brennan, K.; Martin, K.; FitzGerald, S.P.; O'Sullivan, J.; Wu, Y.; Blanco, A.; Richardson, C.; Mc Gee, M.M. A comparison of methods for the isolation and separation of extracellular vesicles from protein and lipid particles in human serum. *Sci Rep* 2020, *10*, 1039, doi:10.1038/s41598-020-57497-7.
20. Huang, K.; Garimella, S.; Clay-Gilmour, A.; Vojtech, L.; Armstrong, B.; Bessonny, M.; Stamatikos, A. Comparison of Human Urinary Exosomes Isolated via Ultracentrifugation Alone versus Ultracentrifugation Followed by SEC Column-Purification. *J Pers Med* 2022, *12*, doi:10.3390/jpm12030340.
21. Jeppesen, D.K.; Fenix, A.M.; Franklin, J.L.; Higginbotham, J.N.; Zhang, Q.; Zimmerman, L.J.; Liebler, D.C.; Ping, J.; Liu, Q.; Evans, R.; et al. Reassessment of Exosome Composition. *Cell* 2019, *177*, 428-445 e418, doi:10.1016/j.cell.2019.02.029.
22. Patel, D.B.; Gray, K.M.; Santharam, Y.; Lamichhane, T.N.; Stroka, K.M.; Jay, S.M. Impact of cell culture parameters on production and vascularization bioactivity of

mesenchymal stem cell-derived extracellular vesicles. *Bioeng Transl Med* 2017, *2*, 170-179, doi:10.1002/btm2.10065.
23. Greenwood, S.K.; Hill, R.B.; Sun, J.T.; Armstrong, M.J.; Johnson, T.E.; Gara, J.P.; Galloway, S.M. Population doubling: a simple and more accurate estimation of cell growth suppression in the in vitro assay for chromosomal aberrations that reduces irrelevant positive results. *Environ Mol Mutagen* 2004, *43*, 36-44, doi:10.1002/em.10207.
24. Pacienza, N.; Lee, R.H.; Bae, E.H.; Kim, D.K.; Liu, Q.; Prockop, D.J.; Yannarelli, G. In Vitro Macrophage Assay Predicts the In Vivo Anti-inflammatory Potential of Exosomes from Human Mesenchymal Stromal Cells. *Mol Ther Methods Clin Dev* 2019, *13*, 67-76, doi:10.1016/j.omtm.2018.12.003.
25. Wang, Y.; Zhang, Z.; Chi, Y.; Zhang, Q.; Xu, F.; Yang, Z.; Meng, L.; Yang, S.; Yan, S.; Mao, A.; et al. Long-term cultured mesenchymal stem cells frequently develop genomic mutations but do not undergo malignant transformation. *Cell Death Dis* 2013, *4*, e950, doi:10.1038/cddis.2013.480.
26. Wu, P.K.; Wang, J.Y.; Chen, C.F.; Chao, K.Y.; Chang, M.C.; Chen, W.M.; Hung, S.C. Early Passage Mesenchymal Stem Cells Display Decreased Radiosensitivity and Increased DNA Repair Activity. *Stem Cells Transl Med* 2017, *6*, 1504-1514, doi:10.1002/sctm.15-0394.
27. Yang, Y.K.; Ogando, C.R.; Wang See, C.; Chang, T.Y.; Barabino, G.A. Changes in phenotype and differentiation potential of human mesenchymal stem cells aging in vitro. *Stem Cell Res Ther* 2018, *9*, 131, doi:10.1186/s13287-018-0876-3.
28. Zhuang, Y.; Li, D.; Fu, J.; Shi, Q.; Lu, Y.; Ju, X. Comparison of biological properties of umbilical cord-derived mesenchymal stem cells from early and late passages: immunomodulatory ability is enhanced in aged cells. *Mol Med Rep* 2015, *11*, 166-174, doi:10.3892/mmr.2014.2755.
29. Digirolamo, C.M.; Stokes, D.; Colter, D.; Phinney, D.G.; Class, R.; Prockop, D.J. Propagation and senescence of human marrow stromal cells in culture: a simple colony-forming assay identifies samples with the greatest potential to propagate and differentiate. *Br J Haematol* 1999, *107*, 275-281, doi:10.1046/j.1365-2141.1999.01715.x.
30. Boregowda, S.V.; Krishnappa, V.; Haga, C.L.; Ortiz, L.A.; Phinney, D.G. A Clinical Indications Prediction Scale Based on TWIST1 for Human Mesenchymal Stem Cells. *EBioMedicine* 2016, *4*, 62-73, doi:10.1016/j.ebiom.2015.12.020.
31. Danaei, M.; Dehghankhold, M.; Ataei, S.; Hasanzadeh Davarani, F.; Javanmard, R.; Dokhani, A.; Khorasani, S.; Mozafari, M.R. Impact of Particle Size and Polydispersity Index on the Clinical Applications of Lipidic Nanocarrier Systems. *Pharmaceutics* 2018, *10*, doi:10.3390/pharmaceutics10020057.
32. Midekessa, G.; Godakumara, K.; Ord, J.; Viil, J.; Lattekivi, F.; Dissanayake, K.; Kopanchuk, S.; Rinken, A.; Andronowska, A.; Bhattacharjee, S.; et al. Zeta Potential of Extracellular Vesicles: Toward Understanding the Attributes that Determine Colloidal Stability. *ACS Omega* 2020, *5*, 16701-16710, doi:10.1021/acsomega.0c01582.
33. Konoshenko, M.Y.; Lekchnov, E.A.; Vlassov, A.V.; Laktionov, P.P. Isolation of Extracellular Vesicles: General Methodologies and Latest Trends. *Biomed Res Int* 2018, *2018*, 8545347, doi:10.1155/2018/8545347.
34. Popovic, M. Routine and novel methods for isolation of extracellular vesicles. *Biologica Serbica* 2019, *41*, 36-43, doi:DOI 10.5281/zenodo.3532082.
35. An, M.; Wu, J.; Zhu, J.; Lubman, D.M. Comparison of an Optimized Ultracentrifugation Method versus Size-Exclusion Chromatography for Isolation of

Exosomes from Human Serum. *J Proteome Res* 2018, *17*, 3599-3605, doi:10.1021/acs. proteome.8b00479.
36. Onodi, Z.; Pelyhe, C.; Terezia Nagy, C.; Brenner, G.B.; Almasi, L.; Kittel, A.; Mancek-Keber, M.; Ferdinandy, P.; Buzas, E.I.; Giricz, Z. Isolation of High-Purity Extracellular Vesicles by the Combination of Iodixanol Density Gradient Ultracentrifugation and Bind-Elute Chromatography From Blood Plasma. *Front Physiol* 2018, *9*, 1479, doi:10.3389/fphys.2018.01479.
37. Yu, L.L.; Zhu, J.; Liu, J.X.; Jiang, F.; Ni, W.K.; Qu, L.S.; Ni, R.Z.; Lu, C.H.; Xiao, M.B. A Comparison of Traditional and Novel Methods for the Separation of Exosomes from Human Samples. *Biomed Res Int* 2018, *2018*, 3634563, doi:10.1155/2018/3634563.
38. Li, K.; Wong, D.K.; Hong, K.Y.; Raffai, R.L. Cushioned-Density Gradient Ultracentrifugation (C-DGUC): A Refined and High Performance Method for the Isolation, Characterization, and Use of Exosomes. *Methods Mol Biol* 2018, *1740*, 69-83, doi:10.1007/978-1-4939-7652-2_7.
39. Willms, E.; Cabanas, C.; Mager, I.; Wood, M.J.A.; Vader, P. Extracellular Vesicle Heterogeneity: Subpopulations, Isolation Techniques, and Diverse Functions in Cancer Progression. *Front Immunol* 2018, *9*, 738, doi:10.3389/fimmu.2018.00738.

Supplemental Figure captions.

Supplemental Figure 1: Expansion characteristics of the canine umbilical cord (CUC) derived mesenchymal stromal cell (MSC) lines. A. Expansion characterized by cumulative population doublings (CPD) versus passage. B. Growth rate characterized by population doubling time (PDT) versus passage. C. PDT versus CPD. A regression line with 95% confidence intervals is shown. Note that this indicates, as suggested in B, that the PDT decreases until passage 7 and then starts to increase. D. CPD versus passage broken down into each of the six MSC lines. Note that one line, CUC 30, had a slower growth rate compared to the other CUC lines.

Supplemental Figure 2: Nanoparticle concentration from nanoparticle tracking analysis (NTA). (A) Particle concentration of extracellular vesicles (EVs) isolates from canine umbilical cord-derived mesenchymal stromal cells (MSCs) from passage 2 to passage 12 (P2 – P12). N differences were detected in the particle counts per mL versus passage for passages 2 through 12. (B) A negative trend was observed when the particle concentration was plotted versus cumulative population doublings (CPD). (C) When the passage data is divided into the early and late passages, Early passages (P2-P5) were significantly different from Late passages (defined as P9-P12) cells. (D) A weak linear association was noted between particle concentration and population doubling time (PDT). (E) EV isolation using ultracentrifugation (UC) was significantly more efficient than size-exclusion chromatography (SEC) isolation. (F) Nanoparticle concentration was equally affected by passage for both SEC and UC. (G-K) Similar differences and trends were observed when the data were plotted by nanoparticles released per MSC. (L) The differences in nanoparticle concentration for each MSC line versus CPD are depicted. The asterisks indicate p-value < 0.05.

Supplemental Figure 3: Size of Extracellular Vesicles measured in three ways. (A) Nanoparticle analysis (NTA) of the size of extracellular vesicles (EVs). (A) NTA-based size measurements comparing EVs from canine umbilical cord-derived mesenchymal stromal cells (MSCs) from the early passage (P2-P5) versus late passage (P9-P12) showed no size differences. (B) A size difference was observed depending upon the EV isolation method: EVs isolated by size-exclusion chromatography (SEC) were significantly larger than EVs isolated by ultracentrifugation (UC). (C & D). Nanoparticle size estimates generated by dynamic light scattering (DLS) showed similar trends compared to NTA, e.g., no effect of passage but significant differences between isolation methods. Note that the size estimates from DLS are much larger than those generated by NTA (A & B) or TEM (E & F). (E & F) Size estimates from transmission electron microscopy. Photomicrographs of EVs are shown in E. Note that TEM micrographs generated from SEC samples were cleaner images than UC. UC samples had many more small particulates that did not have the doughnut appearance of EVs. The calibration bar represents 100 nm. As shown in (F- Top panel), Early passage EVs (median 74.0 nm) were significantly smaller than late passage EVs (98.8 nm). As shown in (F- Bottom panel), no size differences were found between EVs isolation via SEC (median 85.4 nm) and UC (84.0 nm). In contrast, data generated by NTA and DLS, TEM found no size differences between SEC and UC. The asterisks depict p-value < 0.05.

Supplemental Figure 4: Polydispersity index (PDI, A-D) and surface charge (Zeta Potential, E-H) estimated by DLS. PDI: (A) There was no effect of passage on PDI, but an increasing trend in the median was noted. (B) Early passage EVs (P2-P5) displayed an average PDI of 0.46, which is not different from late passage EVs (P9-P12) at 0.48 (Note that the graph depicts median, not average, in the boxplot). (C) EV samples isolated by size-exclusion chromatography (SEC) had a more heterogeneous-sized population distribution than EVs

isolated by ultracentrifugation (UC). EVs separated by SEC had a PDI of 0.55 compared to 0.38 demonstrated by UC-isolated EVs. (D) While PD measured in SEC samples tended not to change with increasing CPD, a more robust trend for PDI to increase with CPD was noted in UC samples. Zeta Potential: No effect of passage was observed overall (E), but significant differences were observed when Early passage was compared to Late passage (F). (G) The surface charge of SEC EV samples was more negative than UC samples. (H) The difference in surface charge between the isolation methods was affected more strongly by the length of culture expansion for UC-isolated EV samples but not by SEC-isolated EV samples.

Table S1. EVs generated per cell

Passage	Mean Previous Passage PDT (hours)	Mean Time in Culture (hours)	Cells Seeded	Mean Population Doublings	Mean Estimated Cell Count
**2**	94.91	115.67	300,000	2.06	$4.59x10^{6}$
**3**	74.67	124.33	300,000	2.19	$4.79x10^{6}$
**4**	70.45	120.67	300,000	1.98	$4.47x10^{6}$
**5**	50.72	110.17	300,000	2.09	$4.64x10^{6}$
**9**	46.41	102.67	300,000	2.26	$4.89x10^{6}$
**10**	39.70	77.0	300,000	2.07	$4.61x10^{6}$
**11**	46.85	82.17	300,000	1.94	$4.41x10^{6}$
**12**	71.72	79.33	300,000	1.66	$3.99x10^{6}$

Table S2: Antibodies used for dot blot

Antibody	Clone	Manufacturer	Catalog Number	Dilution
**CD9**	HI9a	BioLegend	312102	1:400
**CD63**	H5C6	Novus Biologicals	NBP2-42225	1:500
**CD81**	1D6	Novus Biologicals	NB100-65805	1:500
**ALIX**	3A9	Santa Cruz Biotechnology	Sc-53538	1:200
**CD142**	Polyclonal	Bioss Antibodies	BS-4690R	1:300
**β-actin**	2F1-1	BioLegend	643801	1:500
**HRP donkey anti-rabbit IgG**	Polyclonal	BioLegend	406401	1:1000
**HRP goat anti-mouse IgG**	Polyclonal	BioLegend	405306	1:2000

Figure S1

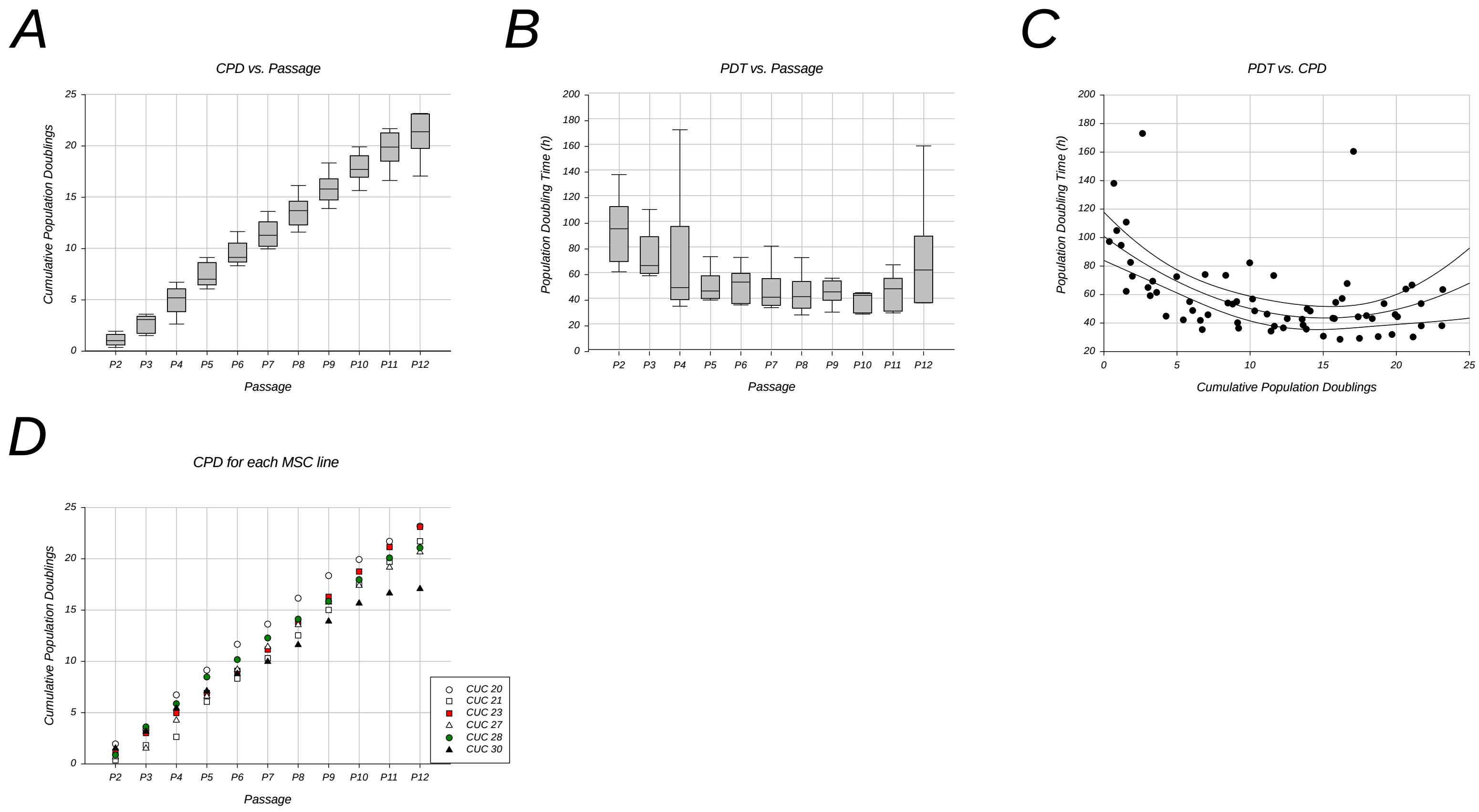
A
CPD vs. Passage
Cumulative Population Doublings
Passage
P2
P3
P4
P5
P6
P7
P8
P9
P10
P11
P12
B
PDT vs. Passage
Population Doubling Time (h)
Passage
C
PDT vs. CPD
Population Doubling Time (h)
Cumulative Population Doublings
D
CPD for each MSC line
Cumulative Population Doublings
Passage
CUC 20
CUC 21
CUC 23
CUC 27
CUC 28
CUC 30

Figure S2

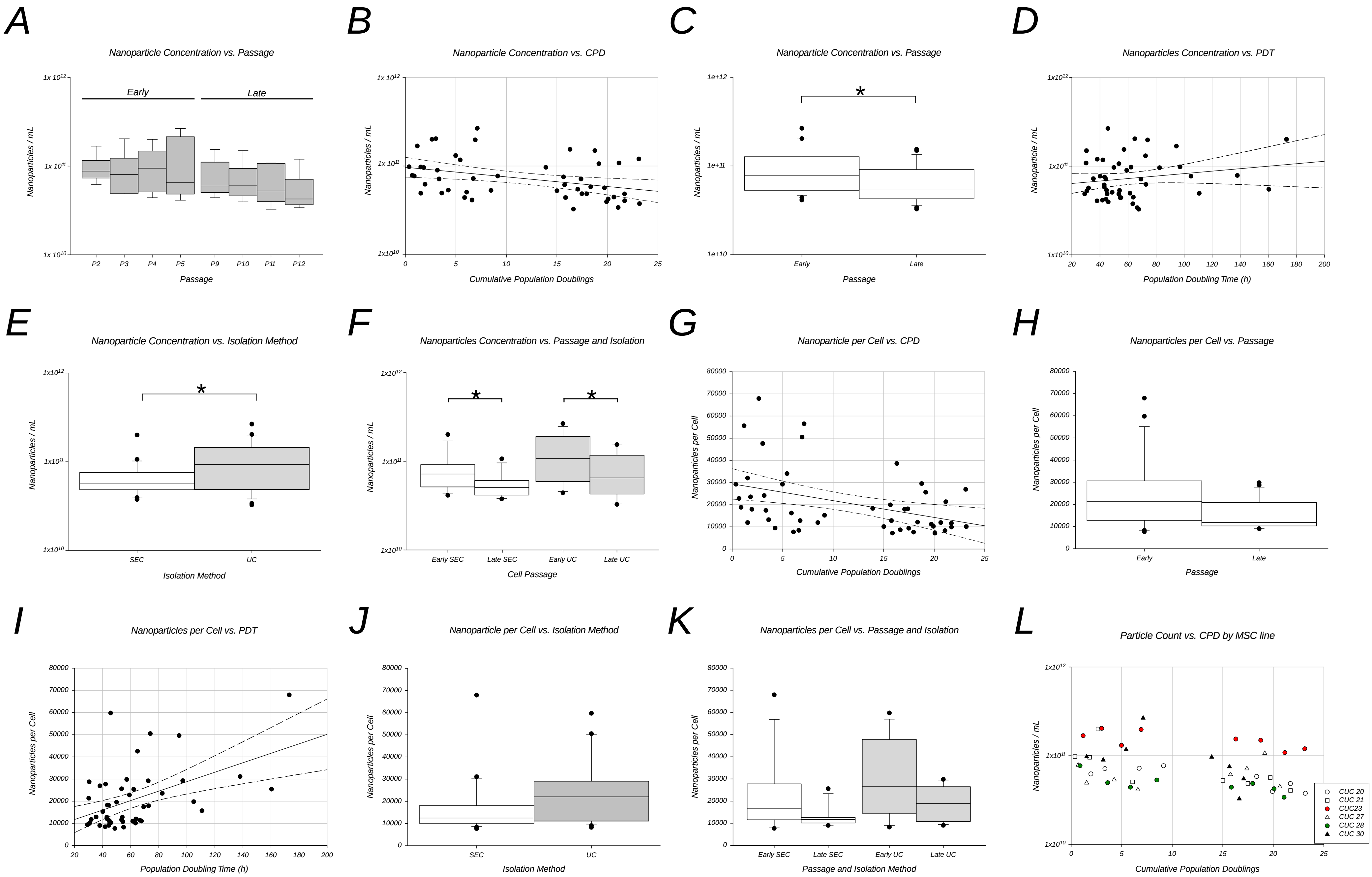

A
Nanoparticle Concentration vs. Passage
Early
Late
Nanoparticles / mL
1x 10^12
1x 10^11
1x 10^10
P2 P3 P4 P5 P9 P10 P11 P12
Passage
B
Nanoparticle Concentration vs. CPD
Nanoparticles / mL
0 5 10 15 20 25
Cumulative Population Doublings
C
Nanoparticle Concentration vs. Passage
*
1e+12
1e+11
1e+10
Early
Late
Passage
D
Nanoparticles Concentration vs. PDT
Nanoparticle / mL
20 40 60 80 100 120 140 160 180 200
Population Doubling Time (h)
E
Nanoparticle Concentration vs. Isolation Method
*
Nanoparticles / mL
SEC
UC
Isolation Method
F
Nanoparticles Concentration vs. Passage and Isolation
*
*
Nanoparticles / mL
Early SEC
Late SEC
Early UC
Late UC
Cell Passage
G
Nanoparticle per Cell vs. CPD
Nanoparticles per Cell
0 10000 20000 30000 40000 50000 60000 70000 80000
Cumulative Population Doublings
H
Nanoparticles per Cell vs. Passage
Nanoparticles per Cell
Early
Late
Passage
I
Nanoparticles per Cell vs. PDT
Nanoparticles per Cell
Population Doubling Time (h)
J
Nanoparticle per Cell vs. Isolation Method
Nanoparticles per Cell
SEC
UC
Isolation Method
K
Nanoparticles per Cell vs. Passage and Isolation
Nanoparticles per Cell
Early SEC
Late SEC
Early UC
Late UC
Passage and Isolation Method
L
Particle Count vs. CPD by MSC line
Nanoparticles / mL
Cumulative Population Doublings
CUC 20
CUC 21
CUC23
CUC 27
CUC 28
CUC 30

Figure S3

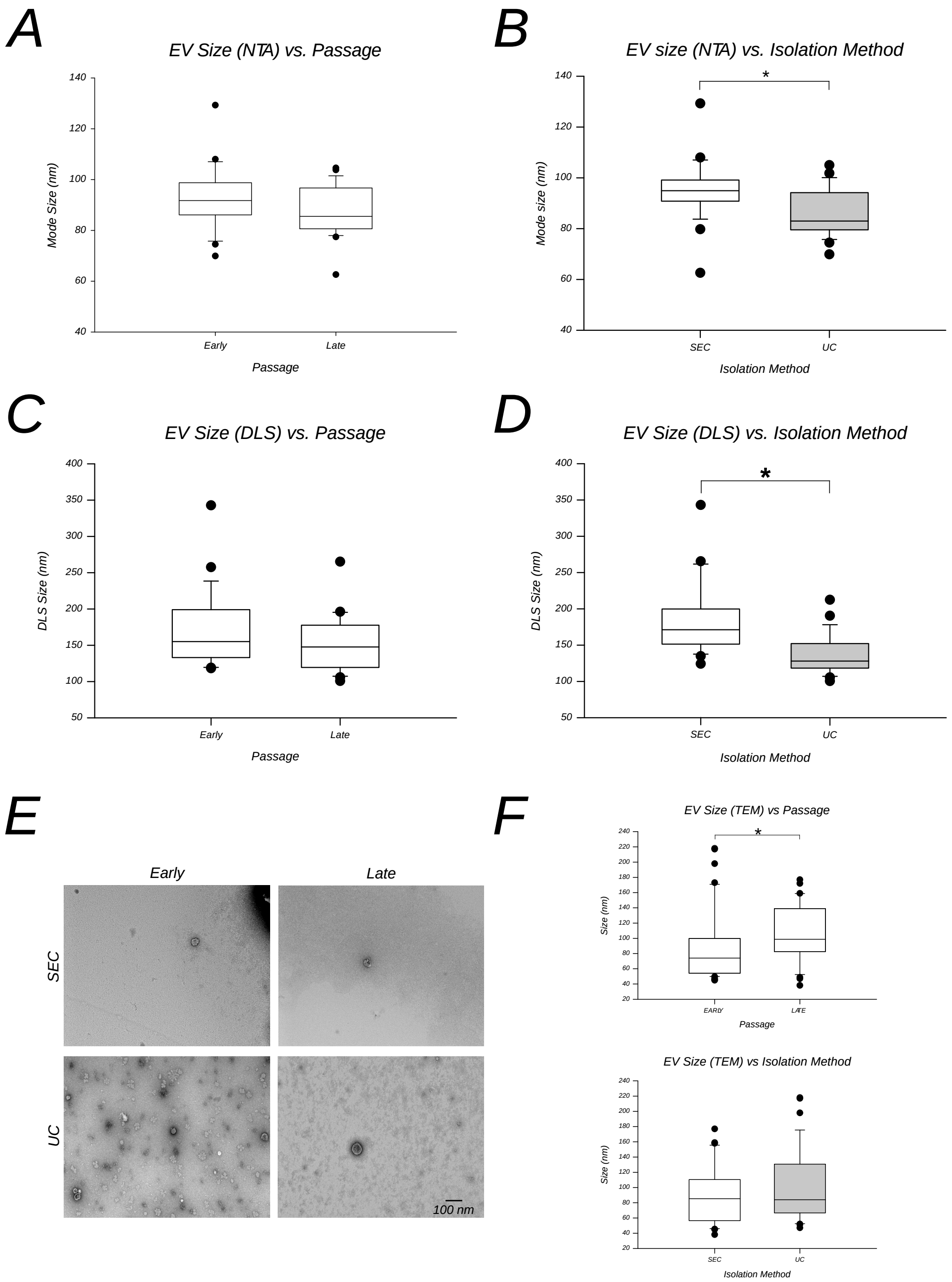
A
EV Size (NTA) vs. Passage
Mode Size (nm)
Early
Late
Passage
B
EV size (NTA) vs. Isolation Method
Mode size (nm)
SEC
UC
Isolation Method
C
EV Size (DLS) vs. Passage
DLS Size (nm)
Early
Late
Passage
D
EV Size (DLS) vs. Isolation Method
DLS Size (nm)
SEC
UC
Isolation Method
E
Early
Late
SEC
UC
100 nm
F
EV Size (TEM) vs Passage
Size (nm)
Passage
EV Size (TEM) vs Isolation Method
Size (nm)
SEC
UC
Isolation Method

Figure S4

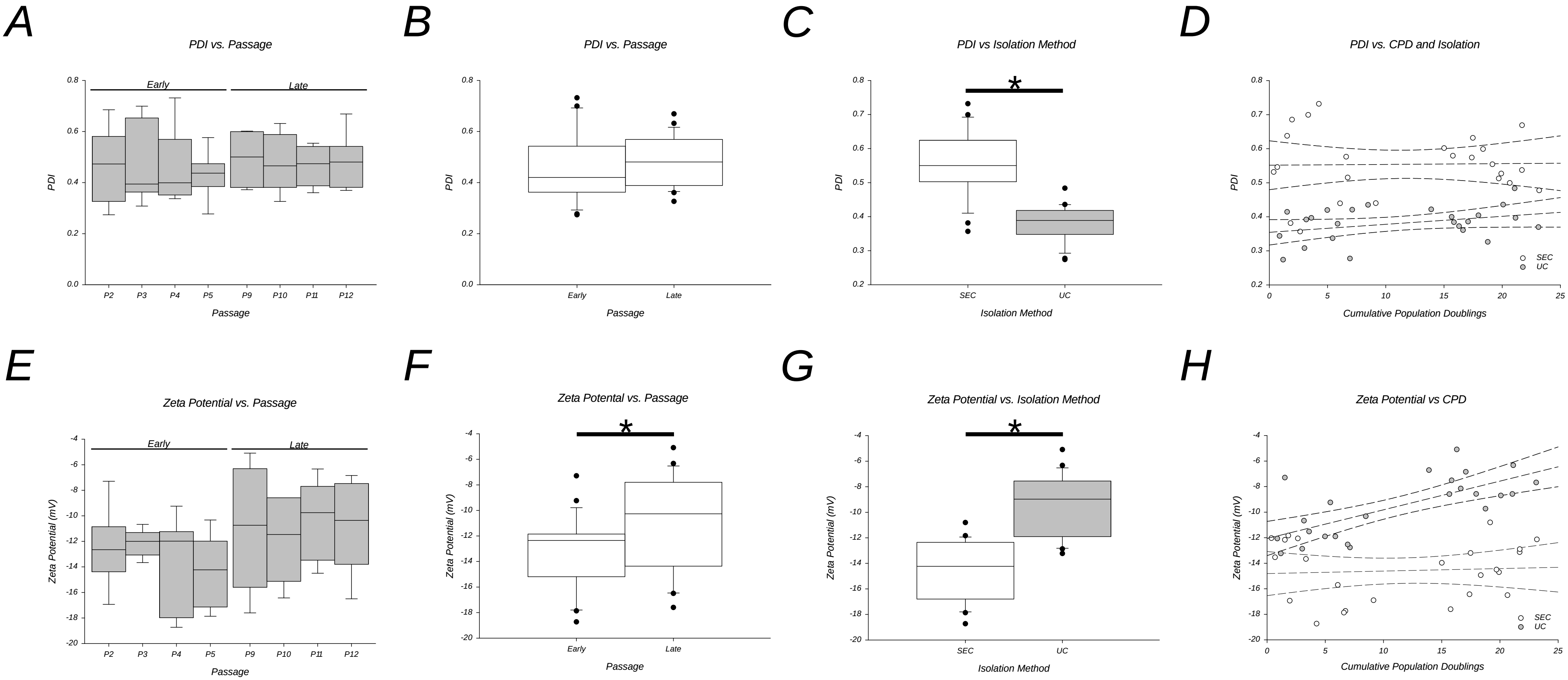
A
PDI vs. Passage
Early
Late
PDI
P2
P3
P4
P5
P9
P10
P11
P12
Passage
B
PDI vs. Passage
PDI
Early
Late
Passage
C
PDI vs Isolation Method
*
PDI
SEC
UC
Isolation Method
D
PDI vs. CPD and Isolation
PDI
SEC
UC
Cumulative Population Doublings
E
Zeta Potential vs. Passage
Early
Late
Zeta Potential (mV)
Passage
F
Zeta Potental vs. Passage
*
Zeta Potential (mV)
Early
Late
Passage
G
Zeta Potential vs. Isolation Method
*
Zeta Potential (mV)
SEC
UC
Isolation Method
H
Zeta Potential vs CPD
Zeta Potential (mV)
SEC
UC
Cumulative Population Doublings
