# Supplementary material for: Procoagulant Activity of Umbilical Cord-Derived Mesenchymal Stromal Cells’ Extracellular Vesicles (MSC-EVs)
Source: Int J Mol Sci. 2023 May 24;24(11):9216. doi: 10.3390/ijms24119216 (PMC10252357; doi:10.3390/ijms24119216)
Supplement: Supplementary file 1 [file ijms-24-09216-s001.zip › CUC Dot Blots.zip!/CUC EV dot blot protocol UC P9 and P10.docx]

**Dot Blot Protocol for EV Antigen Expression**

Protocol Author: Adrienne Wright

Weiss Lab Coles Hall 115

**Table 1: Sample Calculations**

| **Sample #** | **Description** | **Protein Concentration (µg/µL)** | **Dot Volume (µL) for 0.75 µg Protein** |
| --- | --- | --- | --- |
| 1 | Distilled Water (Neg. Control) | -- | 5.0 |
| 2 | Cell Lysate: CUC 020 P9 | 1.46 µg/µL | 0.75/1.46 = 0.51 µL |
| 3 | EVs: CUC 023 P9 | 2.347 µg/µL | 0.75/2.347 = 0.32 µL |
| 4 | EVs: CUC 028 P9 | 0.653 µg/µL | 0.75/0.653 = 1.15 µL |
| 5 | EVs: CUC 030 P9 | 0.6799 µg/µL | 0.75/0.6799 = 1.10 µL |
| 6 | EVs: CUC 023 P10 | 0.5531 µg/µL | 0.75/0.5531 = 1.36 µL |
| 7 | EVs: CUC 028 P10 | 0.5001 µg/µL | 0.75/0.5001 = 1.50 µL |
| 8 | EVs: CUC 030 P10 | 0.5397 µg/µL | 0.75/0.5397 = 1.39 µL |

**Sample Map:**

**Day One Protocol:**

1. Cut PVDF membrane to size needed for sample placement.
2. Activate the PVDF membrane in 99.8% HPLC-grade methanol for 30 minutes at room temperature.
3. Remove methanol and add DI water to keep membrane wet.
4. Place 1 extra thick blot paper in bottom of tray and soak with DI water.
5. Place 1 dry extra thick blot paper on top of the wet paper.
6. Place wet and activated PVDF membrane on top of the blot papers and let water spots dry. The membrane should stay damp! If white spots form, the membrane is too dry and should be reactivated.
7. Dot the calculated volume (Table 1) of samples on membrane according to membrane map (above).
8. Allow the drops to be completely absorbed into the membrane.
9. Remove the blotting papers and rinse the membrane gently with DI water.
10. Add sufficient blocking buffer to the tray to completely cover the membrane.
    a. Blocking Buffer: 5% w/v nonfat dry milk (e.g., 15 g milk in 300 mL 1X TBST buffer)
11. Incubate the membrane in blocking buffer for 30 minutes at room temperature on a slow rocking platform.
12. Dilute primary antibody (calculations in Table 2)
13. Prepare primary antibody solutions by diluting calculated volume (Table 2) of the primary antibody in 10 mL blocking buffer. This can be done directly in labeled trays or using a 15 mL conical tube.
14. Following incubation, carefully cut strips of samples being probed for by each antibody and notch a corner (previously indicated on sample map) to orient.
15. Place strips in diluted antibody and ensure that they are labeled correctly.
16. Incubate overnight at 4°C with gentle rocking.
17. Rocking platform is available in A&P cold room located on the 2^nd^ floor of Coles Hall.

**Second Day Protocol:**

2. Retrieve membranes from cold room.
3. Remove the antibody solutions from the membranes and wash three times with wash buffer.
   a. Wash buffer: 1X TBST buffer (1X Tris-Buffered Saline, 0.1% Tween 20 Detergent), make 10x stock and dilute to 1x as needed. Store at 4°C.
4. Prepare the secondary antibody solution by diluting calculated volume (Table 2) of the secondary antibody in 10 mL of blocking buffer. This can be done separately or by preparing one batch of secondary antibody solution (10mL*number of strips).
5. Incubate for 1 hour at room temperature with gentle rocking.
6. Remove the secondary antibody solution and wash three times with wash buffer. Do not pour the solution directly on the membrane. Leave the membranes in the last wash.
7. Mix equal volumes (5 mL each) of Signaling Reagent A and B (SuperSignal West Femto Substrate, Thermo Fisher, Catalog No. 34095) in reduced light just before imaging.
8. Place individual membrane strip in signaling solution for 10 minutes, protected from light, with gentle rocking.
9. Image strips using the imager in the Cobre Facility of Coles Hall (see imager protocol).

**Table 2: Antibody Calculations**

| **Antibody # (Circle abs used)** | **Antibody Description** | **Manufacturer and Catalog Number** | **Lot #** | **Volume/10mL Blocking Buffer** |
| --- | --- | --- | --- | --- |
| 1 | B-Actin antibody (Clone 2F1-1)  Mouse IgG2b k, Concentration 0.5 mg/mL | BioLegend  643801 | B266258 | 20 µL** |
| 2 | CD9 antibody (Clone HI9a)  Mouse IgG1 k, Concentration 0.5 mg/mL | BioLegend  312102 | B279344 | 50 µL |
| 3 | CD63 antibody (Clone H5C6)  Mouse IgG1 k, Concentration 1 mg/mL | Novus Biologicals  NBP2-42225 | C-2 | 1 – 2 µg/mL  10 – 20 µL |
| 4 | CD81 antibody (Clone 1D6)  Mouse IgG1 k, Concentration 1 mg/mL | Novus Biologicals NB100-65805 | A-8 | 1 – 2 µg/mL  10 – 20 µL |
| 5 | Alix antibody (Clone 3A9)  Mouse IgG1 k, Concentration 200 µg/mL | Santa Cruz Biotech  Sc-53538 | G2920 | 1:200  50 µL |
| 6 | Tissue factor antibody (polyclonal)  Rabbit IgG, Concentration 1 µg/µL | Bioss Antibodies  BS-4690R | AB02241597 | 1:300  33 µL |
| 7 | HRP Donkey anti-rabbit IgG antibody | BioLegend  406401 | B293966 | 10 µL |
| 8 | HRP Goat anti-mouse IgG antibody | BioLegend  405306 | B297976 | 5 µL |

**For B-actin antibody, the binding of the antibody to its target is sensitive to salt concentration. For consistent results, please use TBST buffer for western blotting that contains 0.15 M NaCl as indicated in the BioLegend recommended protocol (see data sheet for more information).
